# The Relation Between Cognition and Interests in Arts Among Chinese Older Adults

**DOI:** 10.1093/geroni/igaa057.1873

**Published:** 2020-12-16

**Authors:** Xia Li, Yuan Fang, Qi Qiu, Shixing Qian

**Affiliations:** Shanghai Mental Health Center, Shanghai, China

## Abstract

This study aims to examine the association between arts related interests and cognition among older Chinese. Data were drawn from 3,243 participants (Mage=71.1, SD=7.8) in the China Longitudinal Aging Study collected in 2011. About 54.4% were female, the average education was eighth grade, 560 interested in music, 86 interested in drawing, and 69 interested in both. Those interested in music or drawing were more likely to enjoy tea and exercise like Taichi, and less likely to smoke or drink (p < 0.01). Those interested in both reported best cognitive function, and those interested in music or drawing had better cognitive function than those without these interests (p< 0.01). However, the difference in cognition between those interested in music and those without diminished after education was controlled. The effect of arts hobbies in cognition among older Chinese remains to be further examined within the context of education and associated lifestyle factors.

